# Effects of school-based physical activity interventions on physical fitness and cardiometabolic health in children and adolescents with disabilities: a systematic review

**DOI:** 10.3389/fphys.2023.1180639

**Published:** 2023-06-09

**Authors:** Marko Manojlovic, Roberto Roklicer, Tatjana Trivic, Rade Milic, Nemanja Maksimović, Roman Tabakov, Damir Sekulic, Antonino Bianco, Patrik Drid

**Affiliations:** ^1^ Faculty of Sport and Physical Education, University of Novi Sad, Novi Sad, Serbia; ^2^ Faculty of Education, Free University of Bozen-Bolzano, Brixen-Bressanone, Italy; ^3^ Sport and Exercise Sciences Research Unit, University of Palermo, Palermo, Italy; ^4^ Faculty of Kinesiology, University of Split, Split, Croatia

**Keywords:** adapted exercise programs, clinical exercise, physical education, vulnerable population, youth

## Abstract

**Background:** This study aimed to examine the influence of school-based physical exercise programs on physical fitness and cardiometabolic health in children and adolescents with disabilities.

**Methods:** Web of Science, Scopus, and PubMed were thoroughly searched to identify relevant investigations. To be included in the systematic review, studies needed to fulfill the following inclusion criteria: 1) performed school-based physical exercise interventions; 2) parameters evaluated referred to health-related physical fitness (HRPF), skill-related physical fitness (SRPF), and cardiometabolic health; 3) a sample of participants consisted of children and adolescents with disabilities; 4) the mean age of respondents was less than 18 years; and 5) were written in the English language.

**Results:** After searching the databases, a total of 474 studies have been identified, 18 of them met the eligibility criteria, and their outcomes were presented. Relating to the respondents’ characteristics, the investigations involved 681 children and adolescents with disabilities, out of which 440 were male and 241 female. Regarding types of physical exercise interventions, the most commonly implemented were combined aerobic and resistance training, aerobic exercise, sports games, adapted high-intensity interval training, as well as sprint interval training. The obtained results unambiguously demonstrated that applied exercise interventions improved HRPF components such as cardiorespiratory fitness, muscular fitness, and flexibility. In terms of the SRPF, agility, balance, coordination, and power were considerably enhanced following the school-based exercise. On the other hand, the influence on indices of body composition and cardiometabolic health is quite controversial. The majority of the available studies did not find favorable effects. Additionally, no adverse events were reported in 7 of 8 research, and adherence to exercise was approximately 92%.

**Conclusion:** School-based physical exercise programs were very efficient in improving HRPF and SRPF in children and adolescents with disabilities, while the evidence concerning the variables of body composition and cardiometabolic health is inconclusive and warrants further investigations.

## Introduction

Disability is defined as a difficulty in functioning at the physical, mental, or sensory level that impairs participation in various aspects of life, including interactions with the external environment (Leonardi et al., 2006). According to the data from the World Health Organization (WHO), there are more than 1,000 million people with disabilities worldwide, which represents approximately 15% of the entire population (World Health Organization, 2015). Of note, available studies indicate a high prevalence of disabilities among children and adolescents (Blackburn et al., 2010; Li et al., 2011; Olusanya et al., 2018; Patrick et al., 2021). For example, a survey conducted in the United Kingdom reported that about 1 million children live with some disabilities (Blackburn et al., 2010). Further, to improve numerous health parameters of disabled children and adolescents, such as physical fitness or cardiometabolic health, the WHO strongly recommended engaging in at least 60 min of moderate-to-vigorous physical activity per day across the week (Bull et al., 2020). Regrettably, most children with disabilities do not meet this recommendation. More specifically, several investigations have shown that more than half of the examined children or adolescents with disabilities were physically active for less than 60 min daily (Wouters et al., 2019; Case et al., 2020). Therefore, engaging in different types of physical exercise, involving those performed in a school environment, appears suitable and indispensable for children and adolescents with disabilities.

Physical fitness consists of two main components: health-related physical fitness (HRPF) and skill-related physical fitness (SRPF) (American College of Sports Medicine, 2013). HRPF refers to health wellbeing and comprises parameters like body composition, cardiorespiratory fitness, muscular fitness (muscle strength and endurance), and flexibility (Caspersen et al., 1985). On the other hand, SRPF relates to performances and contains agility, power, coordination, balance, speed, and reaction time (American College of Sports Medicine, 2013). An increasing body of scientific evidence indicates a trend toward poor levels of physical fitness among children and adolescents with disabilities (Izquierdo-Gomez et al., 2015; Wouters et al., 2020). Regarding body composition, children with disabilities possess higher values of body weight (Neter et al., 2011), body mass index (BMI) (Bertapelli et al., 2016), and waist circumference (Esteban-Figuerola et al., 2021) relative to their typically developing (TD) counterparts. Moreover, Wouters et al. (2020) revealed that cardiorespiratory and muscular fitness were below reference values in children with intellectual disabilities (ID). It is also relevant to highlight that adolescents with Down syndrome had substantially lower levels of SRPF, measured with the motor fitness test, compared to adolescents without Down syndrome (Izquierdo-Gomez et al., 2015). Scientific literature suggests an inverse correlation between physical fitness and cardiovascular diseases (Ortega et al., 2005), mental health issues (Li et al., 2022), and adiposity (Ortega et al., 2013). Thus, various health interventions, such as school-based physical activity programs, are highly desirable to prevent the consequences elicited by exacerbated physical fitness in children or adolescents with disabilities.

A limited number of studies addressed the prevalence of cardiometabolic risk factors in disabled children and adolescents (Wallén et al., 2009; Rimmer et al., 2010; Yamaki et al., 2011; Messiah et al., 2015). All available evidence demonstrates that cardiovascular risk factors are more pronounced in children with disabilities than in their peers without disabilities. More precisely, higher blood pressure has been observed in adolescents with ID and developmental disabilities compared to adolescents in general populations (Rimmer et al., 2010). Furthermore, Yamaki et al. (2011) highlighted significantly elevated blood cholesterol in adolescents with disabilities relative to TD ones. Similarly, systolic blood pressure, triglycerides, and glucose were considerably higher in children marked as disabled, unlike their TD counterparts (Messiah et al., 2015). In addition, the authors emphasized that children and adolescents with disabilities were over three times more likely to have metabolic syndrome than children or adolescents without disabilities (Messiah et al., 2015). Overall, impaired values of cardiovascular risk factors noticeably increased the incidence of cardiovascular disease (Gawlik et al., 2019), type 1 diabetes (Krishnan and Short, 2009), or even premature death (Franks et al., 2010) in children. As in the case of physical fitness, varied lifestyle interventions, including physical exercise within a school setting, are crucial for cardiometabolic health in children or adolescents with disabilities.

Numerous systematic reviews and meta-analyses have explored the impact of school-based exercise on physical fitness (Pozuelo-Carrascosa et al., 2018b; Yuksel et al., 2020; Podnar et al., 2021; Villa-González et al., 2022) and cardiometabolic health (Dobbins et al., 2013; Sun et al., 2013; Pozuelo-Carrascosa et al., 2018a; Duncombe et al., 2022) in non-disabled children and adolescents. There is compelling evidence that school-based physical activity interventions enhance components of physical fitness. Specifically, exercise programs carried out in the school environment positively affected parameters of body composition (Podnar et al., 2021), cardiorespiratory fitness (Pozuelo-Carrascosa et al., 2018b), muscular fitness outcomes (Villa-González et al., 2022), flexibility, and power (Yuksel et al., 2020). Conversely, concerning cardiometabolic health, the available literature is quite inconsistent. Some studies have demonstrated the reduction of cardiovascular risk factors (Pozuelo-Carrascosa et al., 2018a; Duncombe et al., 2022), while others did not find favorable effects (Dobbins et al., 2013; Sun et al., 2013). For instance, Pozuelo-Carrascosa et al. (2018a) evaluated the influence of school-based exercise on markers of cardiometabolic health in children without disabilities. The authors reported positive changes in diastolic blood pressure and fasting insulin. In contrast, Sun et al. (2013) revealed that various forms of physical activities performed within school settings did not alter indices of cardiometabolic health, including triglycerides, low-density lipoprotein cholesterol, total cholesterol, and blood pressure.

In terms of summarizing the literature, available evidence relating to the influence of school-based physical activity interventions on parameters of physical fitness and cardiometabolic health in children and adolescents with disabilities is indeed scarce. Therefore, the objective of this research was to systematically examine the impact of physical exercise conducted in school environments on physical fitness and cardiometabolic health in children and adolescents with disabilities.

## Methods

### Search strategy

Three databases, Web of Science, Scopus, and PubMed, were thoroughly searched from January 2005 to the beginning of December 2022. A Boolean search syntax was employed with the operators “AND” and “OR” and the following keywords: (“school-based physical activity interventions” OR “adapted physical exercise” OR “physical education”) AND (“physical fitness” OR “health-related physical fitness” OR “skill-related physical fitness” OR “cardiometabolic health”) AND (“children” OR “adolescents”) AND (“disabilities” OR “intellectual disabilities” OR “developmental disabilities” OR “physical disabilities”). Reference lists of all relevant studies were manually reviewed to identify additional research. The entire search process was individually conducted by two reviewers (R.R. and T.T.). Any potential disagreement was resolved by discussion or consultation with the first author of this investigation (M.M.). The presented systematic review was performed in line with the recommendations of the Preferred Reporting Items for Systematic Review and Meta-Analyses (PRISMA) statement (Page et al., 2021).

### Eligibility criteria

The studies were considered for inclusion if they fulfilled the following criteria: 1) implemented school-based physical activity interventions (defined as any type of exercise performed exclusively in school settings) (Naylor et al., 2015); 2) evaluated outcomes related to HRPF, SRPF, or indicators of cardiometabolic health; 3) a sample of respondents consisted of children and adolescents with disabilities; 4) the mean age of participants was less than 18 years; 5) were written in the English language. In addition, abstracts, books, non-peer-reviewed journal articles, doctoral theses, systematic reviews and meta-analyses, conference papers, case or brief reports, and study protocols were excluded. At last, investigations that, besides physical exercise, conducted additional interventions, such as nutrition, were not considered for potential inclusion in this systematic review.

### Data extraction

Two authors (R.R. and R.M.) independently extracted data from each of the articles involved in the final analysis. All the retrieved data was entered into a Microsoft Excel template. Information relating to the author’s names and the year of the publication, the study design, as well as the presence of the control group were extracted. In terms of respondents, the extracted data referred to the disability type, sample size, gender, mean age, and obesity status. Overweight or obesity was determined using BMI cut-off values adjusted for the age and gender of the children and adolescents, as proposed by Cole et al. (2000). Regarding exercise programs, data concerning the type of physical activity applied, the duration of the whole intervention, the length of each training session, exercise frequency, intensity, adverse events, and adherence to exercise were retrieved from the research. Finally, all components of HRPF, SRPF, and markers of cardiometabolic health in children and adolescents with disabilities were extracted and presented in the manuscript. All discrepancies between the two reviewers were solved by consensus or after a meeting with the first investigator (M.M.).

### Risk of bias assessment

The Revised Tool for Assessing Risk of Bias in Randomised Trials (RoB 2) (Sterne et al., 2019) and the Risk of Bias in Non-Randomised Studies - of Interventions (ROBINS-I) (Sterne et al., 2016) was applied for quality evaluation in randomized and non-randomized research, respectively. RoB 2 estimates the randomization process, potential deviations from the intended interventions, missing data, measurement of the outcome, and bias in the reported results. Each domain and the overall bias can be rated as “low risk of bias,” “some concerns,” or “high risk of bias.” On the other hand, ROBINS-I assesses confounders, selection of participants, classification of interventions, bias due to deviations from the intended interventions, missing data, outcomes measurement, and the results reported. The domain-level and overall risk of bias are interpreted as “low risk of bias,” “moderate risk of bias,” “serious risk of bias,” “critical risk of bias,” and “no information.” The full description of all domains and the evaluation process for both tools can be found elsewhere (Sterne et al., 2016; Sterne et al., 2019). Two independent reviewers (N.M. and R.T.) rated studies, while contradictions were clarified after consultation with the first author (M.M.).

## Results

### Study selection

**FIGURE 1:**
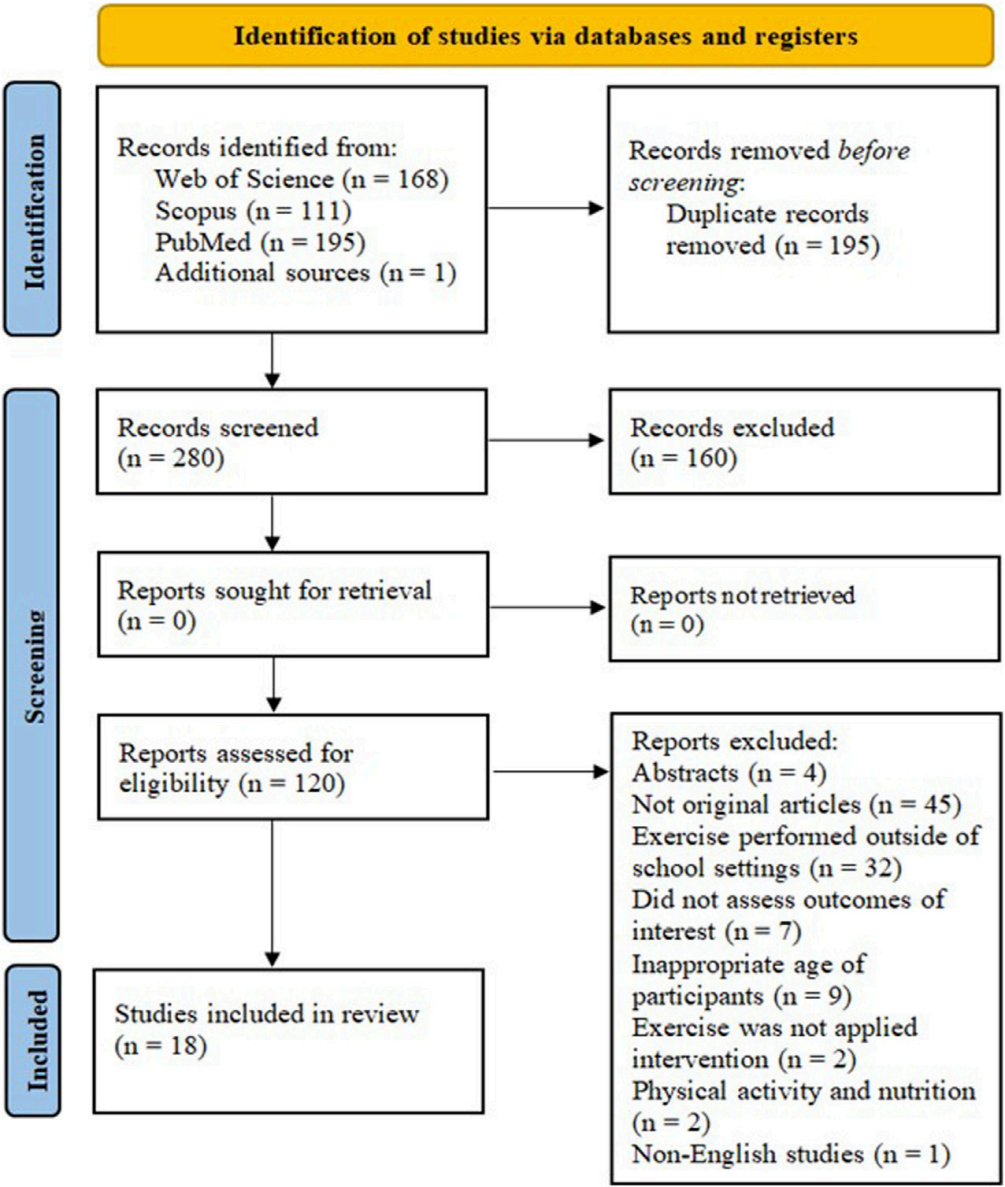
PRISMA flow digram of the study selection process.

Initially, a total of 474 studies were identified via database searches. Checking the reference lists of all relevant records provided one additional study. After eliminating duplicates and screening abstracts and titles, 355 studies were excluded. Of the 120 remaining full-text articles, 102 did not fulfill the eligibility criteria; thus, 18 studies were included in the systematic review. A complete overview of the study selection process is illustrated in Figure 1.

### Description of included studies

All research was published between 2007 and 2022. There were 7 randomized and 11 non-randomized trials (Table 1). The presence of a control group was recorded in 11 out of 18 studies. ID was most common among disability types, followed by autism, Down syndrome, physical disabilities, cerebral palsy, mental disorders, and attention deficit hyperactivity disorder. A total of 681 children and adolescents participated in the studies involved in this systematic review. There were also 440 males and 241 females, with the mean age ranging from 7.4 to 17.5 years old. Respondents were overweight in 4 investigations and obese in 3.

**TABLE 1:** Study design and characteristics of participants.

Study	Study design	Control group	Disability type	Sample size	Gender	Mean age (years)	Obesity status
Bellamy et al. (2020)	Non-RCT	No	ID, autism, ADHD	N = 10	M = 8	10.7	No obesity
					F = 2		
Boer et al. (2014)	RCT	Yes	ID	N = 46	M = 30	17.3 ± 3.0	Overweight
					F = 16		
Davis et al. (2011)	Non-RCT	No	ID	N = 25	M = 16	9.9 ± 1.2	Overweight
					F = 9		
Dodd and Foley (2007)	Non-RCT	Yes	Cerebral palsy	N = 14	M = 10	9.0 ± 2.0	No obesity
					F = 4		
Kao and Wang (2018)	Non-RCT	No	ID	N = 10	M = 10	17.5 ± 0.1	NA
Kocić et al. (2017)	RCT	Yes	ID	N = 50	M = 27	15.8 ± 0.9	No obesity
					F = 23		
Kong et al. (2019)	Non-RCT	Yes	ID	N = 53	M = 45	14.9 ± 3.9	No obesity
					F = 8		
Leahy et al. (2021)	Non-RCT	No	Autism, mental disorders, ID	N = 11	M = 7	17.3 ± 0.7	No obesity
					F = 4		
Ozmen et al. (2007)	RCT	Yes	ID	N = 30	M = 30	11.2 ± 2.0	No obesity
Pan and Mcnamara (2022)	Non-RCT	No	ID, autism, Down syndrome	N = 44	M = 28	15.9 ± 0.4	Overweight
					F = 16		
Salaun et al. (2014)	Non-RCT	No	ID	N = 23	M = 9	15. 1 ± 1.1	Obesity
					F = 14		
Verschuren et al. (2007)	RCT	Yes	Cerebral palsy	N = 68	M = 44	12.2 ± 2.6	No obesity
					F = 24		
Wang et al. (2022)	RCT	Yes	ID, autism, Down syndrome	N = 30	M = 22	14.2 ± 0.5	Overweight and obesity
					F = 8		
Wu et al. (2017)	Non-RCT	Yes	ID, autism, Down syndrome, mental disorders	N = 43	M = 19	17.0 ± 1.4	Obesity
					F = 24		
Xu et al. (2020)	RCT	Yes	Autism, Down syndrome, ID	N = 22	M = 13	7.4	No obesity
					F = 9		
Yu et al. (2022)	RCT	Yes	ID, autism, Down syndrome, ADHD	N = 61	M = 45	15.0 ± 0.6	Obesity
					F = 16		
Zwinkels et al. (2018)	Non-RCT	Yes	Physical disabilities	N = 71	M = 39	13.7 ± 2.9	No obesity
					F = 32		
Zwinkels et al. (2019)	Non-RCT	No	Physical disabilities	N = 70	M = 38	13.4 ± 2.9	No obesity
					F = 32		

Note: ADH, attention deficit hyperactivity disorder; F, female; ID, intellectual disabilities; M, male; NA, not applicable; N, number of participants; RCT, randomized controlled trial.

The description of implemented exercise programs is given in Table 2. Six studies evaluated the effects of combined aerobic and resistance training and 3 studies performed aerobic exercise. Different sports games were carried out in 2 studies, while adapted high-intensity interval training (HIIT) (Zwinkels et al., 2018; Leahy et al., 2021) and sprint interval training (Ozmen et al., 2007; Boer et al., 2014) were also executed. The remaining interventions were an adapted rhythmic gymnastics program (Xu et al., 2020), an adapted basketball training program (Kocić et al., 2017), Tai Chi (Kong et al., 2019), and a frisbee game (Kao and Wang, 2018). The duration of physical activity interventions was between 6 and 60 weeks, with exercise frequency ranging from 1 to 5 sessions per week. The length of individual training sessions was between 20 and 60 min. Exercise intensity was most commonly quantified as a percentage of maximum heart rate (%HRmax) or percentage of heart rate reserve (%HRR).

**TABLE 2:** Exercise description.

Study	Physical activity intervention	Duration (weeks)	Frequency (days/week)	Session length (min)	Exercise intensity	Adverse events	Adherence to exercise
Bellamy et al. (2020)	Combined aerobic and resistance training	16	2	30	Accelerometers	No	86%
Boer et al. (2014)	Sprint interval training	15	2	40	Ventilatory threshold	No	NA
Davis et al. (2011)	Combined aerobic, resistance, and flexibility training	8	5	30	NA	NA	98%
Dodd and Foley (2007)	Treadmill exercise	6	2	30	NA	No	95%
Kao and Wang (2018)	Frisbee game	6	4	40	NA	NA	NA
Kocić et al. (2017)	Adapted basketball training program	8	4	25–35	NA	NA	NA
Kong et al. (2019)	Aerobic dance exercise; Tai Chi	12	2	60	50%–70% HRmax	NA	NA
Leahy et al. (2021)	Adapted HIIT	8	2–3	20–25	70%–80% HRmax	No	NA
Ozmen et al. (2007)	Sprint interval training	10	3	60	60%–80% HRmax	NA	98%
Pan and Mcnamara (2022)	Sport games	60	2	50	NA	NA	NA
Salaun et al. (2014)	Adapted aerobic exercise	30	2	30–50	30%–45% VO₂max	NA	NA
Verschuren et al. (2007)	Combined aerobic and resistance training	32	2	45	NA	Yes	93%
Wang et al. (2022)	Combined aerobic and resistance training	12	2	60	40%–70% HRR	No	97%
Wu et al. (2017)	Combined aerobic and resistance training	12	5	50	70%–80% HRmax	NA	82%
Xu et al. (2020)	Adapted rhythmic gymnastics program	16	3	50	NA	NA	97%
Yu et al. (2022)	Combined aerobic and resistance training	36	2	45	30%–60% HRR	NA	96%
Zwinkels et al. (2018)	Sport games	24	1	45	NA	No	86%
Zwinkels et al. (2019)	Adapted HIIT	8	2	30	NA	No	85%

Note: %, percentage; HIIT, high-intensity interval training; HRmax, maximum heart rate; HRR, heart rate reserve; NA, not applicable; VO₂max, maximal oxygen uptake.

The following parameters of body composition have been assessed: body weight, BMI, waist circumference, the waist-to-hip ratio, the waist-to-height ratio, body fat percentage, and fat-free mass (Table 3). Body weight was reduced in 3 studies, BMI in 4, waist circumference in 3, the waist-to-height ratio in 2, and the percentage of body fat in 4 of 10 trials. Waist-to-hip ratio was unaltered, whereas fat-free mass was decreased in research performed by Wu et al. (2017). An increase in cardiorespiratory fitness was noted in 12 out of 14 investigations. The effects of school-based exercise on muscular fitness have been explored in 12 articles. Five different tests were used for the evaluation of muscle strength and endurance. Favorable effects were obtained in 3 studies that applied the 30 s sit-to-stand test, in all that used 1 min sit-ups, in 3 with the handgrip strength test, in 2 studies that implemented push-ups, and in one that tested the strength of the back muscles (Table 3). Finally, in 3 of the 5 studies, flexibility significantly increased.

**TABLE 3:** Effect of school-based exercise programs on HRPF.

Study	Body composition	Cardiorespiratory fitness	Muscular fitness	Flexibility
Bellamy et al. (2020)	Body weight (kg) ↔	6MWT (m) ↔	NA	NA
	BMI (kg/m^2^) ↔			
	Waist circumference (cm) ↔			
	Wasit-to-height ratio ↔			
Boer et al. (2014)	Body weight (kg) ↔	6MWT (m) ↑	30 s sit-to-stand (repetitions) ↔	NA
	BMI (kg/m^2^) ↔			
	Waist circumference (cm) ↓		Muscle fatigue resistance (s) ↑	
	Body fat (%) ↓			
Davis et al. (2011)	BMI (kg/m^2^) ↔	PACER (laps) ↑	Modified curl-up (repetitions) ↑	Sit-and-reach (cm) ↑
Dodd and Foley (2007)	NA	10MWT (m) ↑	NA	NA
Kao and Wang (2018)	NA	NA	Handgrip strength (kg) ↑	NA
Kocić et al. (2017)	Body weight (kg) ↔	6MWT (m) ↑	NA	NA
	Body fat (%) ↔			
Kong et al. (2019)	BMI (kg/m^2^) ↑	6MWT (m) ↑	1-min sit-ups (repetitions) ↑	Sit-and-reach (cm) ↔
	Waist-to-hip ratio ↔			
	Body fat (%) ↔		Handgrip strength (kg) ↔	
Leahy et al. (2021)	NA	6MWT (m) ↑	30 s sit-to-stand (repetitions) ↑	NA
			Push-ups (repetitions) ↑	
Ozmen et al. (2007)	Body fat (%) ↔	20-MST (laps) ↑	NA	NA
Pan and Mcnamara (2022)	BMI (kg/m^2^) ↓	PACER (laps) ↑	1-min sit-ups (repetitions) ↑	Sit-and-reach (cm) ↑
Salaun et al. (2014)	Body weight (kg) ↔	NA	NA	NA
	BMI (kg/m^2^) ↔			
	Waist circumference (cm) ↓			
	Wasit-to-height ratio ↓			
	Body fat (%) ↓			
Verschuren et al. (2007)	BMI (kg/m^2^) ↔	10-m shuttle run test (min) ↑	30 s sit-to-stand (repetitions) ↑	NA
Wang et al. (2022)	Body weight (kg) ↓	6MWT (m) ↑	30 s sit-to-stand (repetitions) ↑	Sit-and-reach (cm) ↔
	BMI (kg/m^2^) ↓			
	Waist circumference (cm) ↓		1-min sit-ups (repetitions) ↑	
	Wasit-to-height ratio ↔			
	Body fat (%) ↔		Handgrip strength (kg) ↑	
Wu et al. (2017)	Body weight (kg) ↓	NA	1-min sit-ups (repetitions) ↑	NA
	BMI (kg/m^2^) ↓			
	Body fat (%) ↔			
	Fat-free mass (kg) ↓			
Xu et al. (2020)	BMI (kg/m^2^) ↔	10-m PACER run test (cm) ↑	1-min sit-ups (repetitions) ↑	Sit-and-reach (cm) ↑
			Dumbbell press (repetitions) ↑	
			Trunk lift ↑	
Yu et al. (2022)	Body weight (kg) ↓	NA	NA	NA
	BMI (kg/m^2^) ↓			
	Waist circumference (cm) ↓			
	Wasit-to-height ratio ↓			
	Body fat (%) ↓			
Zwinkels et al. (2018)	BMI (kg/m^2^) ↔	10-m shuttle run test (shuttles) ↔	Handgrip strength (kg) ↔	NA
	Waist circumference (cm) ↔			
	Wasit-to-height ratio ↔			
	Body fat (%) ↓			
Zwinkels et al. (2019)	Body weight (kg) ↔	10-m shuttle run test (shuttles) ↑	Handgrip strength (kg) ↔	NA
	BMI (kg/m^2^) ↔			
	Waist circumference (cm) ↔			
	Waist-to-hip ratio ↔			
	Body fat (%) ↔			

Note: ↑, significant increase; ↔, no changes; ↓, significant decrease; %, percentage; BMI, body mass index; cm, centimeters; kg, kilogram; m, meter; min, minute; m^2^, square meter; 20-MST, 20-m shuttle-run test; 10MWT, 10 min walk test; 6MWT, 6 min walk test; NA, not applicable; PACER, progressive aerobic cardiovascular run; s, second.

Agility, balance, coordination, and power were examined as components of SRPF. Agility was improved in 2 research, while for balance and coordination, positive effects have been observed in all investigations. In terms of power, 5 out of 6 studies reported enhancement. Table 4 provides more details concerning variables related to SRPH as well as the tests applied.

**TABLE 4:** Effects of school-based exercise programs on SRPF.

Study	Agility	Balance	Coordination	Power
Kong et al. (2019)	NA	Single-Leg Standing Test (s) ↑	Hopscotch Test—lower body coordination (s) ↓	Vertical jump test (cm) ↑
			Turn-Over-Jars Test—upper body coordination (s) ↓	
Pan and Mcnamara (2022)	NA	NA	NA	Standing long jump test (cm) ↑
Verschuren et al. (2007)	10 x 5-m sprint test (s) ↓	NA	NA	NA
Wu et al. (2017)	NA	Bass dynamic balance (score) ↑	NA	Vertical jump test (cm) ↑
Xu et al. (2020)	NA	NA	NA	Standing long jump test (cm) ↑
Zwinkels et al. (2018)	10 x 5-m sprint test (s) ↔	NA	NA	Standing broad jump test (cm) ↔
Zwinkels et al. (2019)	10 x 5-m sprint test (s) ↓	NA	NA	Standing broad jump test (cm) ↑

Note: ↑, significant increase; ↔, no changes; ↓, significant decrease; cm, centimeters; m, meter; NA, vnot applicable; s, second.

The impact of school-based physical activity interventions on indices of cardiometabolic health is presented in Table 5. Four of the 18 studies investigated the effects of school-based physical activity programs on indices of cardiometabolic health and provided inconsistent results. Cardiovascular parameters were assessed in 4 studies. Systolic blood pressure was reduced in 2 studies, while diastolic blood pressure was decreased in only one. Arterial stiffness remained unchanged following the applied interventions. In terms of metabolic health, values of total cholesterol, triglycerides, high-density lipoprotein (HDL), low-density lipoprotein (LDL), and glucose were estimated in 3 papers. Total cholesterol, triglycerides, and LDL were diminished in one study. Similarly, HDL was augmented in one of the 3 available articles. School-based exercise failed to alter blood glucose in all investigations.

**TABLE 5:** Effects of school-based exercise programs on cardiometabolic health.

Study	Cardiovascular health	Metabolic health
Boer et al. (2014)		Total cholesterol (mg/dL) ↓
	Systolic blood pressure (mmHg) ↓	HDL (mg/dL) ↑
	Diastolic blood pressure (mmHg) ↔	LDL (mg/dL) ↓
		Triglycerides (mg/dL) ↓
		Glucose (mg/dL) ↔
Wang et al. (2022)	Systolic blood pressure (mmHg) ↔	NA
	Diastolic blood pressure (mmHg) ↔	
Zwinkels et al. (2018)		Total cholesterol (mmol/L) ↔
	Systolic blood pressure (mmHg) ↔	HDL (mmol/L) ↔
	Diastolic blood pressure (mmHg) ↔	LDL (mmol/L) ↔
	Arterial stiffness—PWV (m/s) ↔	Triglycerides (mmol/L) ↔
		Glucose (mmol/L) ↔
Zwinkels et al. (2019)		Total cholesterol (mmol/L) ↔
	Systolic blood pressure (mmHg) ↓	HDL (mmol/L) ↔
	Diastolic blood pressure (mmHg) ↓	LDL (mmol/L) ↔
	Arterial stiffness—PWV (m/s) ↔	Triglycerides (mmol/L) ↔
		Glucose (mmol/L) ↔

Note: HDL, high-density lipoprotein; LDL, low-density lipoprotein; PWV, pulse wave velocity; ↑, significant increase; ↔, no changes; ↓, significant decrease; mmHg, millimeters of mercury; m/s, meter per second; mg/dL, milligrams per decilitre; mmol/L, millimoles per liter; NA, not applicable.

### Adverse events and adherence to exercise

Eight of 18 studies provided data relating to adverse events. In 7 investigations, no adverse events were reported. However, one child with cerebral palsy experienced a fracture of the radius in a study conducted by Verschuren et al. (2007). Exercise adherence was recorded in 11 research. The mean adherence to training was approximately 92% for all the studies presented. More details regarding adverse events and adherence to exercise are displayed in Table 2.

### Risk of bias assessment

Solely one of 7 randomized studies had a “low risk of bias,” 3 articles had “some concerns,” and 3 were evaluated as investigations with a “high risk of bias” (Table 6). The largest bias was noted in aspects like the measurement of the outcome, deviations from the intended interventions, and the randomization process. For non-randomized studies, “moderate risk of bias” was recorded in 3 and “serious risk of bias” in 8 research (Table 7). The major sources of bias were a lack of confounders, followed by issues in the measurement of outcomes, deviations from intended interventions, selection of participants, and classification of interventions.

**TABLE 6:** Risk of bias assessment with RoB 2.

Study	Randomization process	Deviations from the intended interventions	Missing outcome data	Measurement of the outcome	Selection of the reported results	The overall risk of bias
Boer et al. (2014)	Low	Low	Low	Some concerns	Low	Some concerns
Kocić et al. (2017)	Some concerns	Some concerns	Low	High	Low	High
Ozmen et al. (2007)	Some concerns	Some concerns	Low	High	Some concerns	High
Verschuren et al. (2007)	Low	Some concerns	Low	Low	Low	Some concerns
Wang et al. (2022)	Low	Low	Low	Low	Low	Low
Xu et al. (2020)	Some concerns	High	Low	High	Low	High
Yu et al. (2022)	Low	Some concerns	Low	Low	Low	Some concerns

Note: high, “high risk of bias”; low, “low risk of bias”.

**TABLE 7:** Risk of bias assessment with ROBINS-I.

Study	Confounding	Selection of participants	Classification of interventions	Deviations from the intended interventions	Missing data	Measurement of outcomes	Selection of the reported results	The overall risk of bias
Bellamy et al. (2020)	Serious	Low	Low	Moderate	Low	Moderate	Low	Serious
Davis et al. (2011)	Serious	Moderate	Moderate	Low	Low	Moderate	Low	Serious
Dodd and Foley (2007)	Serious	Low	Moderate	Low	Low	Serious	Low	Serious
Kao and Wang (2018)	Serious	Low	Moderate	Moderate	Low	Moderate	Low	Serious
Kong et al. (2019)	Serious	Moderate	Low	Low	Low	Serious	Moderate	Serious
Leahy et al. (2021)	Serious	Low	Low	Moderate	Moderate	Low	Low	Serious
Pan and Mcnamara (2022)	Moderate	Low	Moderate	Moderate	Low	Moderate	Low	Moderate
Salaun et al. (2014)	Serious	Moderate	Low	Moderate	Moderate	Serious	Moderate	Serious
Wu et al. (2017)	Moderate	Low	Low	Moderate	Low	Moderate	Low	Moderate
Zwinkels et al. (2018)	Low	Low	Low	Moderate	Low	Serious	Low	Serious
Zwinkels et al. (2019)	Moderate	Moderate	Low	Moderate	Low	Moderate	Low	Moderate

Note: low, “low risk of bias”; moderate, “moderate risk of bias”; serious, “serious risk of bias.”

## Discussion

To the author’s knowledge, this is the first literature review that explored the influence of school-based physical activity interventions on physical fitness and cardiometabolic health in children and adolescents with disabilities. The obtained results unambiguously exhibited that implemented exercise programs induced improvements in most of the components of HRPF and SRPF. However, the effects on parameters of body composition are indeed controversial. Analogous, the evidence regarding the impact on cardiometabolic health markers is inconclusive and warrants further investigations.

In the majority of the presented studies, body composition indices, including body weight, BMI, waist circumference, waist-to-hip ratio, waist-to-height ratio, and body fat percentage, were unaltered following the applied school-based physical activity interventions. The presence of overweight or obesity seems to be a strong predictor of exercise effects on body composition variables. Specifically, implemented interventions were efficient in reducing the mentioned parameters exclusively in investigations conducted on obese or even overweight children and adolescents with disabilities. The presented findings are in agreement with the results obtained in several studies performed on samples of children or adolescents without disabilities (Carrel et al., 2005; Li et al., 2014; Seabra et al., 2020; Cao et al., 2022). For instance, the school-based soccer program has been proven to be very effective in reducing waist circumference, waist-to-hip ratio, and percentage of body fat in overweight children (Seabra et al., 2020). Moreover, HIIT executed within school settings successfully decreased body weight, BMI, body fat percentage, and fat mass in obese children (Cao et al., 2022). Overall, the effects of school-based exercise programs on body composition parameters in disabled children and adolescents, as well as the mechanisms responsible for the outcomes, remain unclear and require deeper and more thorough examinations in further studies. Additionally, fat-free mass was evaluated in only one of the available studies, and positive effects were not obtained. Hence, the following research should focus on the impact of school-based exercise programs on other indices of body composition, such as muscle mass or bone parameters.

In terms of effects on the remaining components of HRPF, the results shown are quite unequivocal. More precisely, physical exercise carried out within the school environment represents a powerful training tool for the enhancement of cardiorespiratory fitness in children and adolescents with disabilities. An increase in cardiorespiratory fitness has been observed irrespective of test or exercise interventions applied, characteristics of participants, and composition of training variables. Furthermore, robust evidence indicates that exercise programs induce improvements in variables of muscular fitness. Flexibility was also increased in 3 out of 5 accessible investigations. The results obtained referring to the mentioned components of physical fitness are supported by the studies conducted on TD children or adolescents. Various types of physical activity interventions carried out in school surrounding were efficient in enhancing cardiorespiratory fitness (Kriemler et al., 2010; Meyer et al., 2014; García-Hermoso et al., 2020; Kolle et al., 2020), muscular fitness (Martínez-Vizcaíno et al., 2020; Katsanis et al., 2021; Sinđić et al., 2021), and flexibility (Ardoy et al., 2011; Gallotta et al., 2017).

Similarly, as with HRPF, all aspects of SRPF, including agility, balance, coordination, and power, were improved after the implementation of various school-based physical activity interventions. Concerning children and adolescents without disabilities, the available literature is also consistent. Of note, school-based HIIT positively influenced agility in adolescents 10–19 years old (da Silva Bento et al., 2021). Moreover, the karate program elicited enhancement in balance, estimated with the Y-balance test, in apparently healthy children (Pinto-Escalona et al., 2021). At last, values of power have been magnified as a result of participation in several types of physical exercise carried out within school settings (Trajković et al., 2020; Lee et al., 2021). Since the SRPF is strongly linked with performance, improvements in agility, balance, coordination, and power likely contribute to successful participation in numerous team and individual sports performed outside of school by children and adolescents with disabilities. In other words, the enhancement of SRPF components following the school-based exercise programs can enable children or adolescents with disabilities to be involved in different types of physical exercises as well as other life aspects completely or approximately equally with their TD peers. Finally, considering that a limited number of studies evaluated the impact of school-based exercise programs on the parameters of SRPF, more investigations are warranted to verify the presented findings.

As previously highlighted, increasing physical fitness in children and adolescents has significant health implications. More specifically, improvements in cardiorespiratory fitness, muscular fitness, and agility are correlated with countless health benefits, such as enhancement of mental health, reducing the risk of cardiovascular disease, and preservation of skeletal health, respectively (Ortega et al., 2008). Therefore, school-based physical activity programs can be considered clinically meaningful in high-risk and vulnerable populations like disabled children and adolescents.

Relating to the effects of school-based physical activity programs on cardiometabolic health, evidence is quite inconsistent. For example, Boer et al. (2014) explored the influence of sprint interval training on parameters of cardiovascular and metabolic health in overweight adolescents with ID. The authors revealed that applied intervention reduced systolic blood pressure, whilst diastolic blood pressure was unchanged. Regarding metabolic health, total cholesterol, triglycerides, and LDL cholesterol were decreased, HDL cholesterol increased significantly, and glucose was not altered. Furthermore, Zwinkels et al. (2018) examined the effects of sports games on cardiometabolic health markers in non-obese children with physical disabilities. The implemented exercise program did not provoke favorable effects concerning any of the indices of cardiometabolic health. Analogous, another study conducted by Zwinkels et al. (2019) analyzed the impact of adapted school-based HIIT in non-obese children and adolescents with physical disabilities. Only systolic and diastolic blood pressure were decreased, while other variables of cardiovascular or metabolic health remained unaltered. Based on the highlighted facts, it appears that the exercise effects are primarily determined by the implemented physical activity intervention but also by the potential presence of overweight or obesity in respondents. Most importantly, some adapted versions of the high-intensity interval activities could be deemed appropriate for improvements in cardiometabolic health in children and adolescents with disabilities. Several studies investigated school-based exercise effects on indices of cardiometabolic health in children and adolescents without disabilities. HIIT induced favorable effects relating to total cholesterol and triglycerides (Solera-Martinez et al., 2021), HDL cholesterol (Meng et al., 2022), and glucose (Bauer et al., 2022). Sprint interval training and aerobic exercise positively changed LDL cholesterol and blood pressure, respectively (McMurray et al., 2022; Salus et al., 2022). Given that a restricted number of articles addressed the influence of school-based physical activity interventions on the parameters of cardiometabolic health in children and adolescents with disabilities, all drawn conclusions must be interpreted with some caution, and additional investigations are needed.

### Adverse events and adherence to exercise

Keeping in mind that in 7 out of 8 research, no adverse events have been reported, school-based physical activity programs can be considered safe for children and adolescents with various disabilities. Moreover, available studies indicate high adherence to exercise. Actually, disabled children and adolescents attended more than 90% of the physical activity sessions during the investigations. Thus, it should be inferred that the examined population favors exercising within school environments. Most importantly, there is quite firm evidence that school-based physical exercise programs are feasible for the vulnerable population, such as children or adolescents with disabilities. At last, studies that examined adverse events and adherence to exercise in a sample of TD children and adolescents are in line with the findings from the presented research (Lubans et al., 2010; Martin-Smith et al., 2019). For example, no injuries or other adverse events were reported, with training attendance of about 80% in adolescents that performed resistance training in school settings (Martínez-Vizcaíno et al., 2014).

### Strengths and limitations

`The presented systematic review has several strengths that must be highlighted. First, most of the included studies are actual and recently published (within the last 5 years). Second, almost all of the investigations likely had enough statistical power to detect predicted training effects, which is truly rare in studies from the field of sports sciences. Third, the outcomes of this investigation have relevant health implications and are useful for physical education teachers and other stakeholders whose expertise is closely related to the clinical exercise.

On the other hand, certain limitations need to be acknowledged. The main criticism refers to the high and serious risk of bias observed in the randomized and non-randomized trials, respectively. Thus, higher-quality investigations are warranted in the future. In addition, the absence of a control group was noted in some articles. In terms of the gender of the participants, the majority of the research was conducted on a sample of male children or adolescents with disabilities. Hence, more studies are needed with female participants. Finally, due to the high heterogeneity among the tests used for the evaluation of physical fitness and cardiometabolic health parameters, as well as the design of the analyzed studies, meta-analysis was not an appropriate solution.

## Practical implications

The main findings of this investigation are particularly useful for physical education teachers and suggest that the implementation of different types of physical activities, including combined aerobic and resistance training, aerobic exercise, sports games, and adapted high-intensity interval activities, has relevant health implications. Specifically, school-based exercise programs with a duration of 12 weeks or more, performed 2–3 times per week, with the length of individual training sessions of approximately 40 min at a low to moderate intensity expressed as %HRR or %HRmax are clinically meaningful for children and adolescents with disabilities. Moreover, due to the absence of adverse events in most of the studies, physical education teachers are encouraged to continue to conduct an exercise in school environments in high-risk populations like disabled children and adolescents. In order to improve cardiometabolic health parameters in classes with overweight and obese children or adolescents, it is recommended to implement some adapted types of high-intensity interval activities.

## Conclusion

School-based physical activity interventions are very efficient in improving SRPF and HRPF in children and adolescents with varied disabilities. However, the influence on components of body composition and cardiometabolic health remains unclear and requires further examination. Additionally, school-based exercise programs have been proven safe and favorable for disabled children or adolescents. Physical education teachers need to continue to promote exercise in school settings to maintain or enhance health in high-risk and vulnerable populations such as children and adolescents with disabilities.

## Data Availability

The original contributions presented in the study are included in the article/supplementary material, further inquiries can be directed to the corresponding author.
